# A Japanese Case of Lenz‐Majewski Syndrome With a Novel *PTDSS1* Variant

**DOI:** 10.1002/mgg3.70112

**Published:** 2025-06-17

**Authors:** Yasuko Kobari, Non Miyata, Jun Takayama, Naoya Saijo, Tomohisa Suzuki, Shigeo Kure, Atsuo Kikuchi, Gen Tamiya, Takumi Takizawa

**Affiliations:** ^1^ Department of AI and Innovative Medicine Tohoku University Graduate School of Medicine Sendai Japan; ^2^ Department of Pediatrics Gunma University Graduate School of Medicine Maebashi Japan; ^3^ Department of Biochemistry and Cell Biology National Institute of Infectious Diseases Tokyo Japan; ^4^ Department of Rare Disease Genomics Tohoku University Graduate School of Medicine Sendai Japan; ^5^ Department of Pediatrics Tohoku University Graduate School of Medicine Sendai Japan; ^6^ Miyagi Children's Hospital Sendai Japan

**Keywords:** congenital cutis laxa, Lenz‐Majewski syndrome, phenotypic spectrum, *PTDSS1* gene

## Abstract

**Background:**

Lenz‐Majewski syndrome (LMS) is a rare genetic disorder characterized by osteosclerosis, intellectual disability, characteristic facies, and distinct craniofacial, dental, cutaneous, and distal‐limb anomalies. Mutations in the *PTDSS1* gene, which encodes one of the phosphatidylserines (PS) synthase enzymes, PSS1, have been identified as causative in LMS patients. These mutations make PSS1 insensitive to feedback inhibition by PS levels.

**Methods:**

Whole genome sequence (WGS) was performed on a patient with congenital *cutis laxa* and her parents. PS synthase activity was analyzed in *PTDSS1* mutant cDNA clones to evaluate functional alterations.

**Results:**

A 5‐year‐old girl presented with congenital skin wrinkles and was initially diagnosed with congenital *cutis laxa*. She had bilateral inner ear hypoplasia, bilateral low‐frequency hearing loss, attention‐deficit/hyperactivity disorder, and mild intellectual disability. Physical examination revealed protruding ears, frontal bossing, and dental malalignment. A *de novo* heterozygous missense variant in the *PTDSS1* gene, c.284G>A (p. Arg95Gln) was identified by WGS. Functional analysis indicated increased PS synthase activity, supporting the pathogenicity of this variant.

**Conclusions:**

The patient's *cutis laxa* and facial features were consistent with LMS, though radiographic findings did not reveal the characteristic sclerosing bone dysplasia reported in previous cases. This observation suggests that LMS may have a broader phenotypic spectrum than previously recognized.

## Graphical abstract

A Japanese girl with a de novo PTDSS1 R95Q variant showed mild sclerosing bone dysplasia. Functional and structural analyses revealed intermediate PS synthesis activity compared to previously reported variants, suggesting a broader phenotypic spectrum of LMS.

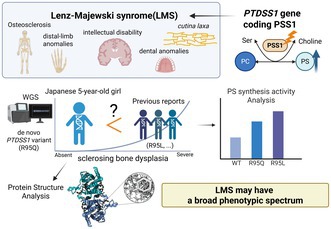

## 1 Introduction

Lenz‐Majewski syndrome (LMS, OMIM:151050) is a rare genetic disorder characterized by osteosclerosis, intellectual disability, characteristic facial features, distinct craniofacial features, and unique craniofacial, dental, skin, and distal‐limb anomalies. Mutations in the *PTDSS1* gene (OMIM: 612792), encoding one of the phosphatidylserines (PS) synthase enzymes, PSS1, have been identified as causative in LMS patients. These gain‐of‐function mutations render PSS1 insensitive to feedback inhibition by PS levels (Online Mendelian Inheritance in Man, OMIM, n.d.).

PS is an essential phospholipid for mammalian cell growth (Vance and Tasseva 2013). The PS formation in mammalian cells occurs through the exchange of L‐serine with the choline moiety of phosphatidylcholine (PC) or the ethanolamine moiety of phosphatidylethanolamine (PE). In Chinese hamster ovary cells, this is catalyzed by at least two enzymes, PSS1 and PSS2 (Tomohiro et al. 2009; Leventis and Grinstein 2010).

Kuge et al. previously found that replacing Arg95 of PSS 1 with a lysine residue affects feedback inhibition by PS, indicating that Arg95 of PSS 1 is a critical amino acid residue for regulating PSS 1 (Kuge et al. 1998; Ohsawa et al. 2004). To date, 20 cases have been described in the literature, and 12 cases have been molecularly confirmed (Braham 1969; Lenz and Majewski 1974; Kaye et al. 1974; Robinow et al. 1977; Gorlin and Whitley 1983; Saraiva 2000; Nishimura et al. 1997; Wattanasirichaigoon et al. 2004; Dateki et al. 2007; Sousa et al. 2014; Mizuguchi et al. 2015; Whyte et al. 2015; Tamhankar et al. 2015; Piard et al. 2018; Murtaza et al. 2018; Afifi et al. 2019; Maden Bedel et al. 2024). We report the 13th case of LMS diagnosed by molecular genetic testing, who shares the Arg95 amino acid substitution previously described in two cases (Piard et al. 2018; Afifi et al. 2019), but presents with milder bone sclerosis. This case provides an opportunity to examine the genotype‐phenotype correlation of different amino acid substitutions at the Arg95 residue of PSS1.

## 2 Materials and Methods

### 2.1 Whole Genome Sequence and Computational Prediction of Variant Pathogenicity

Whole genome sequencing analysis was performed as previously described (Takayama et al. 2021). The pathogenicity of the identified SNVs, insertions, deletions, SVs, and CNVs was evaluated using several in silico prediction tools, including SIFT, PolyPhen‐2, and CADD. Additionally, we visually inspected the data using Integrative Genomics Viewer (IGV) to check for structural variations.

### 2.2 DNA Transfection of HeLa Cells and Evaluation of PS Synthetic Activity Using [14C]Serine

HeLa cells (1 × 10^5^ cells/well) were seeded in a 24‐well plate and transfected the following day with 0.5 μg of each *PTDSS1* plasmid. After 24 h, [^14^C] serine (final concentration 1 μCi/mL) was added, and cells were cultured for 2 h. Lipids were then extracted using the Bligh‐Dyer method and separated by TLC. [^14^C] PS and [^14^C] PE were detected and quantified by autoradiography.

### 2.3 Prediction of Protein Structure in *PTDSS1* Mutants

Protein structure data were obtained from the Protein Data Bank (PDB ID: 9B4E).

## 3 Results

### 3.1 Case Report

A Japanese 5‐year‐old girl presented with skin wrinkles since birth and was diagnosed with congenital *cutis laxa*. She was born at term (38 weeks and 1 day) with a birth weight of 2226 g and a length of 44 cm. At 10 months, a skin biopsy revealed a loss of elastic fibers and tears in the middle dermis, confirming the diagnosis as congenital *cutis laxa*. She began walking independently at 1 year and 4 months and spoke her first words at 1 year and 8 months. She was later diagnosed with attention‐deficit/hyperactivity disorder (ADHD) and mild intellectual disability. Her parents were non‐consanguineous, and her younger sibling was healthy.

**FIGURE 1:**
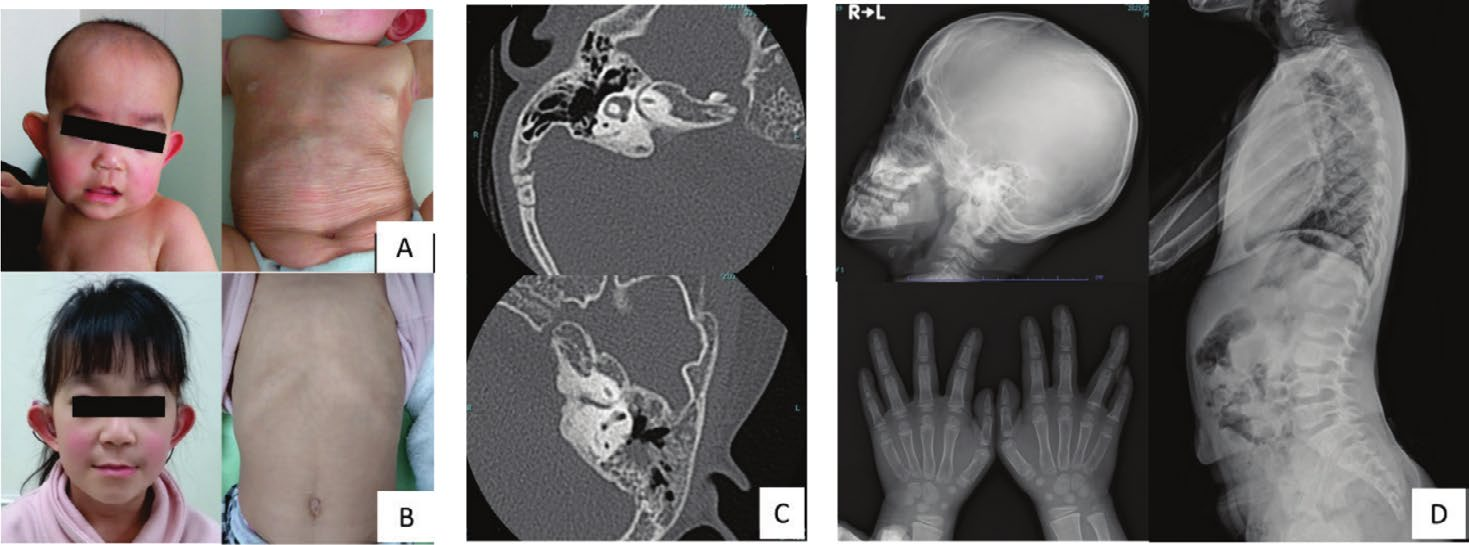
Clinical photographs and radiological imaging of facial, skin, and bone features. (A) 10‐month‐old (B) 6‐year‐old (C) Inner ear hypoplasia is shown by a temporal bone CT scan at 2 years old. (D) Short middle phalanges of hands, no signs of bone sclerosis in the whole body.

At presentation, her height was −1.0 SD, and her body weight was −0.4 SD. Physical examination showed protruding ears, frontal bossing, dental malalignment, generalized skin laxity, and bilateral low‐frequency hearing loss (Figure 1).

Trio whole genome analysis identified a *de novo* heterozygous missense variant in the *PTDSS1* gene (NM_014754.3), c.284G>A (p. Arg95Gln). ClinVar lists two entries for this variant (rs1131691429) with conflicting interpretations of pathogenicity: one as ‘pathogenic’ and another as ‘uncertain significance’. Previous studies suggest that the Arg95 of PSS1 is a critical amino acid residue for feedback inhibition by PS (Ohsawa et al. 2004). As shown in Table 1, *in silico* analysis indicated high pathogenic scores. Based on evaluation according to the ACMG/AMP 2015 guidelines, the variant was classified as likely pathogenic, supported by criteria PS2, PS3, PM1, PM2, and PM6.

**TABLE 1:** *In Silico* analysis results of Arg95Gln variant.

Variant	SIFT score	PolyPhen‐2 score	CADD Score	Mutation taster	AlphaMissense	Classification
R95Q	0.003 (Deleterious)	1.0 (Damaging)	34	1 (Deleterious)	0.99 (Pathogenic)	Likely Pathogenic

*Note:* The Arg95Gln variant is considered likely pathogenic, as multiple *in silico* analyses indicated high pathogenic scores.

In response to the reviewer's suggestion, we reanalyzed the whole genome sequencing data of the proband, carefully examining single‐nucleotide variants (SNVs), insertions, deletions, copy number variants (CNVs), and structural variants (SVs). We also visually inspected intronic regions using the Integrative Genomics Viewer (IGV) to ensure that no potentially pathogenic intronic variants or structural rearrangements, such as inversions or translocations, were overlooked. This comprehensive reanalysis did not reveal any additional pathogenic variants, and no evidence supporting a dual diagnosis was found. We did not perform karyotyping in this case, as we believe it was not necessary based on the comprehensive results obtained from whole genome sequencing.

### 3.2 Previous Case Review

To date, 12 cases of LMS confirmed through molecular genetics have been reported, our case is the 13th case (Table 2) (Piard et al. 2018; Afifi et al. 2019; Maden Bedel et al. 2024).

**TABLE 2:** Literature review of PTDSS1 gene variants in patients with LMS.

Exon number	Exon3			Exon7						Exon9			
Author	Kobari	Piard	Afifi	Tamhankar	Sousa	Piard	Sousa	Piard	Whyte	Chrzanowska	Saraiva	Wattanasirichaigoon	Bedel
Published year	2025	2018	2019	2015	2014	2018	2014	2018	2015	1989	2000	2004	2024
Mutation PTDSS1	c.284G>A	c.284G>T	c.284G>T	c.785G>T	c.794T>C	c.794T>C	c.805C>T	c.806C>T	c.829T>C	c.1058A>G	c.1058A>G	c.1058A>G	c.1058A>G
	p.R95Q	p.R95L	p.R95L	p.R262I	p.L265P	p.L265P	p.P269S	p.P269L	p.W277R	p.Q353R	p.Q353R	p.Q353R	p.Q353R
Sex	F	M	M	M	F	F	M	F	F	M	F	F	F
Origin	Japanese	ND	Egyptian	Indian	Czech	Czech	Kurd‐Turkish	Romanian	ND	Polish	Portuguese	Thai‐Chinese	Turkish
Birth‐genstational age (weeks)	38	41	39	Term	39	Term	38	38	36	41	37	38	39
Length, cm (SD)	44 (−2.0)	46 (−3)	51 (+0.5)	ND	44 (−4)	44 (−3.5)	ND	51 (+0.3)	ND	52 (−1.4)	45 (−1.3)	46 (−1.3)	ND
Weight, g (SD)	2226 (−1.6)	2310 (−2.2)	2250 (−2.5)	2480	2700 (−1.3)	2700 (−1.8)	2740 (−0.7)	2570 (−1.4)	2570 (5%tile)	2550 (−2.5)	3470 (+1.3)	2100 (< −2)	2700 (−1.38)
Head circumference, cm (SD)	32.5 (−0.4)	32 (−3)	35 (−0.5)	ND	32 (−2.2)	32 (−3)	ND	31 (−2.5)	ND	35 (+0.1)	33 (0)	30 (< −2)	ND
Postnatal growth ‐age, ‐years, ‐months (m)	5	24	15 m	7	9	9	2	3 m	5.5 m	36	18	11.5	9
Hight, cm (SD)	103 (−1.0)	123.4 (−8.6)	65 (−4)	103 (−3.6)	84 (−10)	84 (−9)	72.3 (−4)	ND	58.6 (−2.3)	128.6 (−7.8)	135 (−4.8)	98 (−7)	90 (−7.1)
Weight, kg (SD)	16.9 (−0.4)	33.8 (−5)	5.8 (−4.5)	18 (−2.0)	10 (−5)	10.1 (−8.7)	8.2 (−3)	ND	4.6 (−4.6)	34 (3.5)	39.5 (−2.9)	17 (−3)	14.5 (−4.2)
Head circumference, cm (SD)	ND	54 (−2)	44.5 (−1.3)	46.5	32 (−2.2)	45.4 (−5)	43.3 (−4)	34 (−5.9)	41.6	53 (−2.7)	52.5 (−1.6)	50.5 (−2)	48 (−2.8)
Intellectual disability	Moderate	Severe	Severe	Severe	Severe	Severe	Mod‐severe	Mild	Mild	Borderline	Mild‐Modertate	Severe	Severe
Speech inpairment	delayed	a few words	ND	babble	2 words	absent	ND	ND	ND	good	sentences	absent	absent
**Craniofacial features**													
Delayed closure of fontanelles	−	+	+	+	+	+	+	+	+	ND	+	+	+
Broad/prominent forehead	+	+	+	+	+	+	+	+	+	+	+	+	+
Hypertelorism/telencanthus	+	+	+	+	+	+	+	+	+	+	+	+	+
Large ears	+	−	−	+	+	+	+	+	+	+	+	+	−
Oligodontia	+	ND	+	ND	ND	+	ND	ND	+	ND	ND	ND	ND
Dental enamel hypoplasia	−	ND	+	+	+	ND	ND	ND	ND	+	+	ND	ND
Macroglossia/protruded tongue	−	−	−	+	−	−	+	−	−	−	+	+	−
Macrostomia	+	+	+	+	+	+	+	+	+	+	+	+	−
Prognathism/retrognathia	+	+	+	+	+	+	+	+	+	+	+	+	+
Thin vermilion od the lips	+	+	+	+	+	+	ND	+	+	+	+	+	+
Sparse hair	+	+	+	+	+	+	+	+	+	+	+	+	+
Loose atrophic skin/cutis laxa	+	+	+	+	+	+	+	+	+	+	+	+	+
Prominent cutaneous veins	+	+	+	+	+	+	+	+	+	+	+	+	+
Joint laxity	−	+	+	+	+	+	+	ND	+	+	+	+	+
**Distal limbs**													
Brachydactyly, more severe post‐axially	−	+	+	+	+	+	+	+	+	+	+	+	+
Proximal symphalangism	−	+	+	+	+	ND	+	ND	+	+	+	+	+
Cutaneous syndactyly	−	+	+	+	+	+	+	+	+	+	+	+	+
**Radiological features**													
Skull and ventebra progressive sclerosis	−	+	ND	+	+	+	+	ND	+	+	+	+	+
Turricephaly	+	+	+	+	+	+	ND	ND	+	ND	ND	+	−
Progressive diaphyseal hyperostosis	−	+	+	+	+	+	+	ND	+	+	+	+	+
Diaphyseal undermodeling	−	+	+	+	+	+	+	ND	+	+	+	+	+
Meta/epiphyseal radiolucency	−	+	+	+	+	+	+	ND	+	+	ND	+	ND
Broad clavicles and ribs	−	+	ND	−	+	ND	+	ND	+	+	+	+	+
Short/absent middle phalanges	+	+	+	+	+	+	+	ND	+	+	+	+	+
Phalangeal synostosis	−	+	+	−	+	ND	+	ND	+	+	+	−	ND
Metacarpal synostosis	−	+	−	−	−	ND	−	ND	+	−	−	−	+
Kyphoscoliosis	−	+	ND	−	+	ND	+	ND	−	ND	ND	ND	ND
Abnormal/delayed skeletal maturation	−	+	+	+	+	+	+	ND	+	+	+	+	ND
**Brain imaging**	**MRI**	**ND**	**MRI**			**MRI**	**CT nl at 15 m**	**ND**	**CT**	**ND**	**MRI**	**CT, MRI, MRV**	**MRI**
Diffse cortical atrophy	−		+	+	+	+			−		−	+	−
Hydrocephalus/enlarged ventricles	−		+	−	+	+			−		−	+	−
Pituitary hypoplasia	−		−	−	+	+			ND		−	+	−
Leucoencephalopathy	−		−	−	−	−			ND		+	+	−
Small central spinal canal	−		−	−	−	−			ND		−	+	−
Vascular anomalies	−		−	−	−	−			ND		−	+	−
Dysgenesis of the corpus callosum	−		−	−	−	−			−		+	−	+
**Others**													
Cranial nerve palsy	−	−	−	−	−	−	−	−	−	−	−	+	−
Cleft palate	−	−	−	−	−	−	−	−	−	−	−	+	−
Hearing loss	+	+	−	−	−	−	ND	−	+	+	+	−	−
Ophthalmological problems	−	−	−	Optic atrophy	−	−	−	−	+	Hypermetropia	Small iris	Entropion	−
Inguinal Hernia	−	−	−	−	−	−	+	−	+	−	+	−	−

Abbreviations: ND, Not defined; nl, no lesion.

**FIGURE 2:**
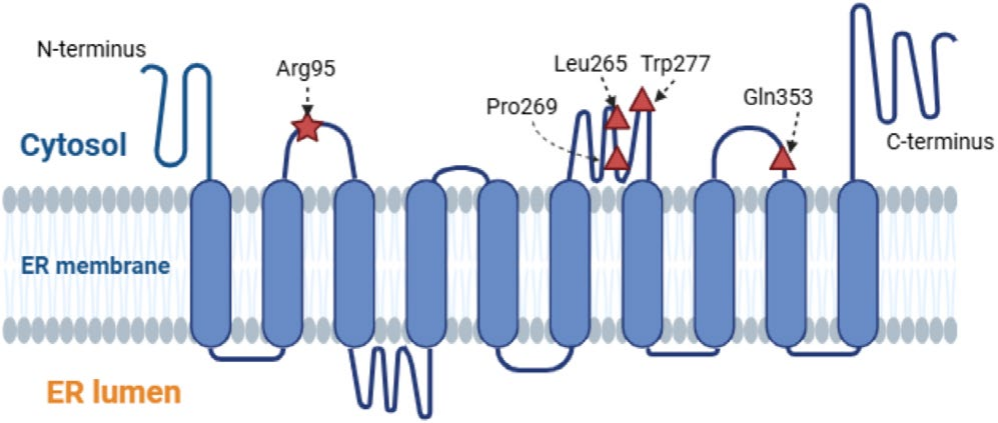
Structural representation of PTDSS1 with locations of LMS variants.

The sites of LMS mutations are shown in the 10‐transmembrane protein structure of PTDSS1 (Miyata and Kuge 2021), which is oriented toward cytosol, as is also the case for Arg95. (Figure 2). Two LMS cases with the heterozygous missense variant c.284G>T (p.Arg95Leu) in the *PTDSS*1 have been reported (Piard et al. 2018; Afifi et al. 2019). Our case displayed atypically mild osteosclerosis compared to previous reports. Two additional mild LMS cases have been reported in Japan before (Nishimura et al. 1997; Dateki et al. 2007), but genotyping was unavailable. Functional analysis was performed to investigate potential genotype‐phenotype correlations.

The structure of PTDSS1 is shown to have a 10‐transmembrane topology, with Arg95 and all previously reported variant sites (Leu265, Pro269, Trp277, Gln353) located on the cytosolic side.

### 3.3 PS Synthetic Activity Analysis and Protein Structure Prediction

**FIGURE 3:**
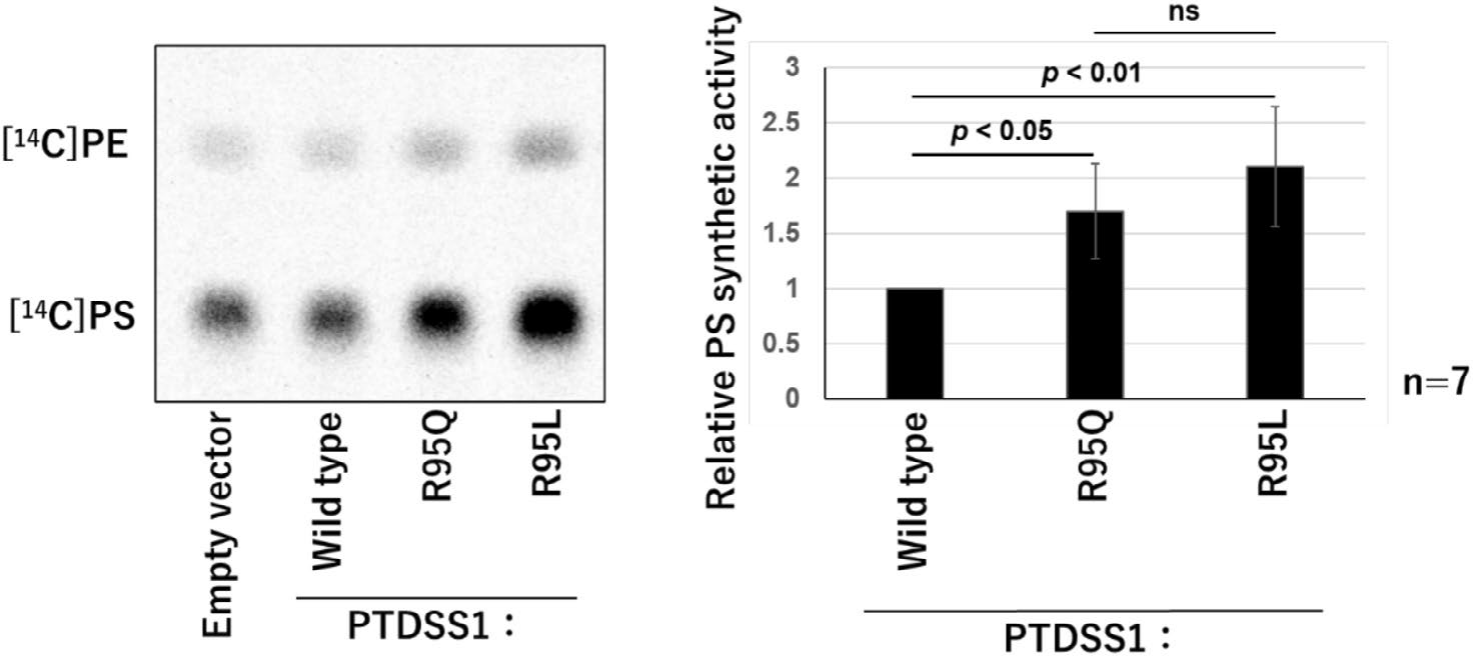
Comparison of PS synthesis activity by amino acid substitution of Arg95 in HeLa cells.

PS synthase activity in *PTDSS1* mutant cDNA clones (Arg95Gln and Arg96Leu) was significantly higher than in the wild‐type cDNA transfectant (Figure 3). The Arg95Gln mutant was confirmed as pathogenic, but there was no significant difference compared to the Arg95Leu mutant.

The PS synthase activity of Arg95Gln (R95Q) and Arg95Leu (R95L) transfectants was significantly increased compared to wild type. This supports the pathogenic nature of the R95Q variant. No statistically significant difference was observed between R95Q and R95L. Bars represent mean values; error bars show standard deviation (SD). Statistical analysis was performed using Tukey's test.

**FIGURE 4:**
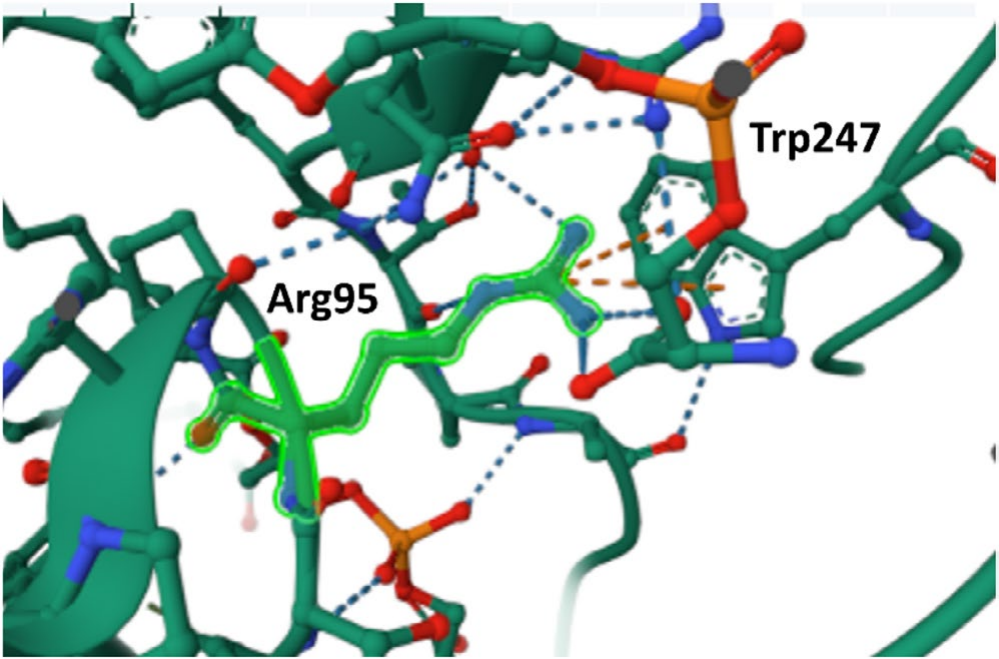
Predicted 3D structure of *PTDSS1* showing Arg95 position based on PDB data.

Protein structure analysis using PDB data revealed two cation‐π interactions between Arg95 and Trp247, critical for local structural stability (Figure 4). Substitution of Arg95 with Leu (a non‐polar residue) or Gln (a neutral, polar residue) weakens this interaction due to the loss of positive charge, compromising the interaction with Trp247's π‐electrons.

Arg95 residue shown in PDB, with an orange dashed line indicating a cation‐π interaction with Trp247.

## 4 Discussion

Phosphatidylserine (PS) is the most abundant negatively charged glycerophospholipid in eukaryotic membranes, functioning as a key component of the cell membrane (Tomohiro et al. 2009; Leventis and Grinstein 2010). PS plays essential roles in various cellular processes, including intracellular signaling (Leventis and Grinstein 2010), apoptosis (Fadok et al. 1992), blood coagulation (Bevers and Williamson 2016), cholesterol transport (Vance 2018), and viral infection (Birge et al. 2016). It is also critical in maintaining the structural integrity of nerve cell membranes and myelin (1). In eukaryotic cells, PS is predominantly localized to the cytosolic leaflet of the plasma membrane and is also found in late endosomal compartments (Van Meer et al. 2008). During apoptosis, PS translocates from the inner to the outer leaflet of the plasma membrane, a movement thought to be pivotal for signaling cell death (Fadok et al. 1992).

Gain‐of‐function mutations in the *PTDSS1* gene, which increase PS production, have been linked to LMS (Leventis and Grinstein 2010; Bevers and Williamson 2016; Vance 2018; Kimura and Kimura 2021; Yeung et al. n.d.). These mutations are known to disrupt the organization of the actin cytoskeleton in osteoclasts, contributing to the characteristic skeletal abnormalities observed in LMS (Glade and Smith 2015; Kim et al. 2014).

Piard et al. (2018) suggested considering LMS in the differential diagnosis of neonatal cases with congenital *cutis laxa*. Polydactyly and facial dysmorphism are useful features for diagnosis; notably, in LMS, congenital *cutis laxa* tends to improve with age, contrasting with other syndromes presenting congenital *cutis laxa*. LMS can also be distinguished from other forms of congenital *cutis laxa* by its association with severe skeletal dysplasia.

Compared to previously reported cases of LMS, the mild osteosclerosis observed in our case might be due to the specific position of the Arg95 residue and differences in the properties of the substituted amino acids, which may differentially impact the function of the PSS1 protein. Although PS exchange activity assays with Arg95Leu and Arg95Gln substitutions showed no statistically significant difference, Arg95Leu tended to exhibit higher activity than Arg95Gln. This difference may contribute to the phenotypic variation, although further evidence would be needed to confirm this hypothesis.

Notably, the same R95 residue was altered in patients reported by Piard et al. (Piard et al. 2018) and Afifi et al. (2019), both of whom harbored the R95L variant and presented with more severe skeletal phenotypes. Differences in the molecular characteristics of leucine and glutamine may influence the structure or function of the PSS1 protein, thereby contributing to phenotypic variability. Furthermore, although no additional pathogenic variants were identified in our patient, we cannot entirely exclude the possibility of a dual diagnosis, as suggested by the reviewer. These findings highlight the importance of considering allelic heterogeneity and potential genetic modifiers when interpreting genotype–phenotype relationships in PSS1‐related conditions.

Long et al. (2024) previously determined the PSS1 structure in which two PSS1 molecules form a homodimer with two phospholipid‐binding pockets (sites 1 and 2) located around PH4 on the cytosolic leaflet of each PSS1 protomer. Phospholipid density was observed in each positively charged cavity, stabilizing two cation‐πinteractions (Arg95/Trp247 and Arg336/Trp272). In site 1, PS binds via hydrophilic interactions, while in site 2, more extensive interactions between PS and PSS1 were observed through MD simulations. Mutating Arg95 to alanine increased the PS2 production, likely due to the disruption of these binding sites. This finding suggests that PSS1 may regulate its activity in response to cytoplasmic PS levels through these binding site (Long et al. 2024). This PS binding site not only includes Arg95 but also encompasses previously reported hot spots, such as Leu265Pro and Pro269Ser, located in the same region. These findings support the hypothesis that PSS1 likely senses cytosolic PS levels and regulates its activity through these two PS binding sites. The structural analysis suggests substituting Arg95 with Leu or Gln may destabilize the protein. Reducing PS binding stability could impair PS level sensing in the cytoplasm, leading to loss of regulatory control over PS activity and contributing to the observed clinical phenotype.

These findings suggest a phenotypic spectrum in LMS, potentially influenced by PS binding may contribute to the range of clinical manifestations observed in LMS.

## 5 Conclusion

Our patient's *cutis laxa* and facial features were consistent with LMS, but the absence of sclerosing bone dysplasia suggests a broader phenotypic spectrum for the LMS condition.

## Author Contributions

Yasuko Kobari conceived the study plan and wrote the manuscript. Non Miyata was responsible for conducting the PSS1 activity assays and contributed to analyzing and interpreting these results. Gen Tamiya, Jun Takayama, Shigeo Kure, Atsuo Kikuchi, Naoya Saijo and Tomohisa Suzuki carried out genetic studies. Takumi Takizawa performed the clinical investigation and supervised the study.

## Ethics Statement

The Tohoku University Research Ethics Committee approved this study on October 4th, 2022, under approval number 2022‐1‐611 (Registration No. 27284). Written informed consent was obtained from all participants.

## Consent

Informed consent was obtained from the family to publish this case report and clinical images.

## Conflicts of Interest

The authors have received financial support from Astellas Pharma Inc. and declare this as a competing interest.

## Data Availability

All data generated or analyzed during this study are included in this article.
